# Influenza vaccine uptake in juvenile idiopathic arthritis: a multi-centre cross-sectional study

**DOI:** 10.1007/s00431-024-05552-0

**Published:** 2024-04-15

**Authors:** Despoina Maritsi, Foteini Dasoula, Amit Ziv, Maša Bizjak, Barbora Balažiová, Matija Matošević, Mehmet Yildiz, Noa Alpert, Lovro Lamot, Ozgur Kasapcopur, Tomáš Dallos, Yosef Uziel, Natasa Toplak, Merav Heshin-Bekenstein

**Affiliations:** 1 Second Department of Pediatrics, P. & A. Kyriakou Children’s Hospital, National and Kapodistrian University of Athens, Athens, Greece; 2 Pediatric Rheumatology Unit, Department of Pediatrics, Meir Medical Center, Kfar Saba, Israel; 3 School of Medicine, Tel Aviv University, Tel Aviv, Israel; 4 Department of Allergology, Rheumatology and Clinical Immunology, University Children’s Hospital and Medical Faculty, Ljubljana, Slovenia; 5 Department of Paediatrics, Comenius University Medical School in Bratislava, National Institute of Children’s Diseases, Bratislava, Slovakia; 6 Department of Pediatrics, University of Zagreb School of Medicine, Zagreb, Croatia; 7 Cerrahpasa Medical School, Istanbul University-Cerrahpasa, Istanbul, Turkey; 8 Department of Pediatrics, University Hospital Center Zagreb, Zagreb, Croatia; 9 Pediatric Rheumatology Service, Dana Dwek Children’s Hospital, Tel Aviv Sourasky Medical Center, 6 Weizmann Street, Tel Aviv, 6423906 Israel

**Keywords:** Influenza vaccine, Juvenile Idiopathic Arthritis (JIA), Vaccination, Uptake, Children

## Abstract

While most countries provide safe and effective influenza vaccines for at-risk groups, influenza vaccine coverage among children with rheumatic diseases remains uncertain. This study investigated influenza vaccination rates in children with juvenile idiopathic arthritis (JIA) during the 2019–2020 season and assessed the knowledge and attitudes of caregivers of children with JIA regarding influenza vaccination. The secondary aims were to identify barriers to vaccination and explore strategies to improve vaccination rates. A multi-centre, cross-sectional anonymous survey was conducted in 7 countries during the 2019–2020 influenza season to assess the uptake history of influenza vaccination. Among 287 participants, only 87 (30%) children with JIA received the influenza vaccine during the 2019–2020 season. Children who were more likely to be vaccinated were those with systemic juvenile idiopathic arthritis (sJIA), a history of previous vaccination and those aware of the vaccination recommendations. Conversely, children who previously experienced adverse vaccine-related events reported the lowest uptake. The primary reason for non-vaccination was lack of awareness about the necessity of influenza vaccination.

*  Conclusion*: Despite variations among countries, the uptake of influenza vaccines remains low in children with JIA. Improving awareness among families about the importance of influenza vaccination may increase vaccination rates in children with rheumatic diseases.

**Table:** 

**What is Known:** *• Rheumatic children are at increased risk for influenza infection due to immunosuppressive therapy and immune dysregulation.* *• Influenza vaccine is formally recommended to children with rheumatic diseases.*	
**What is New:** *• This multicentre study showed that influenza vaccine uptake rates remain suboptimal among children with Juvenile Idiopathic Arthritis despite formal recommendations.* *• Factors like previous experience with vaccination and information provided by medical professionals via different ways play essential roles in increasing vaccination rates and can contribute to improved health outcomes for these vulnerable children.*

**What is Known:**

*• Rheumatic children are at increased risk for influenza infection due to immunosuppressive therapy and immune dysregulation.*

*• Influenza vaccine is formally recommended to children with rheumatic diseases.*

**What is New:**

*• This multicentre study showed that influenza vaccine uptake rates remain suboptimal among children with Juvenile Idiopathic Arthritis despite formal recommendations.*

*• Factors like previous experience with vaccination and information provided by medical professionals via different ways play essential roles in increasing vaccination rates and can contribute to improved health outcomes for these vulnerable children.*

## Introduction

Influenza is a widespread, infectious disease affecting 5–10% of adults and 20–30% of children worldwide, annually [1]. Influenza infections contribute to high rates of hospitalizations with associated morbidity and mortality [2]. Children with rheumatic diseases (RD) are at increased risk for infections due to immunosuppressive therapy and possible immune dysregulation, and are prone to severe influenza complications [3].

Seasonal influenza vaccination is the most effective measure to prevent or attenuate infection with influenza and its associated complications. The European League Against Rheumatism (EULAR) recommendations for vaccination in children with RD were published in 2011 and updated in 2023 [4], and include annual administration of influenza vaccination for this target group during the fall and winter seasons [5]. National recommendations for immunization of immunocompromised patients are available in some countries [6] and safe and effective influenza vaccines are available in most. Despite available recommendations and vaccines, reports from some countries suggest that vaccine coverage in patients with RD is much lower than among healthy individuals [7–9]. The rates of adherence to vaccination guidelines and influenza vaccine coverage among target groups is unknown.

The primary objectives of this study were to assess the influenza vaccination rate in children with Juvenile Idiopathic Arthritis (JIA), to investigate the knowledge and perceptions of caregivers regarding influenza vaccination, and to identify barriers and facilitators that could be used to promote vaccine uptake.

## Materials and methods

This multi-centre, cross-sectional study was conducted in Croatia, Cyprus, Greece, Israel, Slovakia, Slovenia, and Turkey during the 2019–2020 influenza vaccination season, among parents of children with JIA. The study was conducted by the Paediatric Rheumatology European Society (PReS) Vaccination Working Party. The survey tool was translated into the native languages of the participating countries and was pilot-tested and revised for clarity and understanding. It included 25 closed-ended questions concerning influenza vaccination uptake history during the 2019–2020 season, before the COVID-19 pandemic; knowledge and perceptions regarding influenza vaccination; and demographics and clinical data regarding JIA. Caregivers of children with JIA attending outpatient clinics were invited to complete the anonymous questionnaire. The response rate was 100%.

### Statistics

Descriptive statistics were used to compute absolute and relative frequencies. Associations were evaluated using the Chi-square test of independence to determine the significant factors associated with caregivers’ perceptions regarding the flu vaccine. Data are presented as n (%) for categorical variables and as mean ± standard deviation (SD) for continuous variables. P-values < 0.05 were considered statistically significant. SPSS v.20 (IBM Corp., Armonk, NY) was used to analyse the data.

## Results

A total of 287 JIA caregivers of 287 JIA patients were surveyed across the 7 countries that participated in the study (Table 1). Their mean age was 41.6 ± 7.3 years and 217 (75.6%) were female. Most participants were employed (72%, n = 207), married (82.5%, n = 237) and had achieved a high level of education (50.9%, n = 146). The most common diagnosis of the children was oligoarticular JIA (28.9%, n = 83), followed by polyarticular JIA (20.9%, n = 60) and systemic JIA (9.1%, n = 26), while 28.6% (n = 82) of caregivers did not know their child’s JIA subtype. The mean age of the children was 10.5 ± 4.8 years, with median disease duration of 4 years (interquartile ratio 2–7). Among the 287 patients, 205 (71.4%) were receiving systemic treatment. Of these 205 children, 97 (47%) were receiving methotrexate, 36 (17.5%) TNF inhibitors, and 38 (18.5%) both methotrexate and TNF inhibitors; the rest were receiving corticosteroids, or other drugs at the time of the survey.

**Table 1:** Demographics, clinical data, vaccination history, and their effect on influenza vaccine uptake in the 2019–2020 season (N = 287)

**Variable**	**Level**	**N (%)**	**Vaccinated the child against flu in 2019–2020 season N (%)**		
			**Yes** n = 87 (30.3%)	**No** n = 200 (69.7%)	*P*-value*
**Country of origin**	Croatia	33 (11.5)	0	33 (100)	**0.00**
	Cyprus	22 (7.7)	3 (13.6)	19 (86.4)	
	Greece	65 (22.6)	46 (70.8)	19 (29.2)	
	Israel	62 (21.6)	26 (41.9)	36 (58.1)	
	Slovakia	46 (16)	0	46 (100)	
	Slovenia	43 (15)	7 (16.3)	36 (83.7)	
	Turkey	16 (5.6)	5 (31.3)	11 (68.7)	
**Employment status**	Employed	207 (72)	53 (25.7)	154 (74.3)	**0.01**
	Unemployed	30 (10.5)	16 (53.3)	14 (46.7)	
	Self-employed	40 (14)	16 (40)	24 (60)	
	Social scheme	10 (3.5)	2 (20)	8 (80)	
**Education level**	Elementary	26 (9.1)	8 (30.8)	18 (69.2)	0.59
	Secondary	115 (40)	31 (27)	84 (73)	
	Tertiary	146 (50.9)	48 (32.9)	98 (67.1)	
**Marital status**	Married	237 (82.5)	80 (33.8)	157 (66.2)	0.06
	Divorced	28 (9.8)	4 (14.3)	24 (85.7)	
	Separated	6 (2.1)	1 (16.7)	5 (83.3)	
	Widowed	6 (2.1)	2 (33.3)	4 (66.7)	
	Single-parent	10 (3.5)	0	10 (100)	
**Child’s principal diagnosis**	Oligo-JIA	83 (28.9)	26 (31.3)	57 (68.7)	**0.00**
	Poly-JIA	60 (20.9)	22 (36.7)	38 (63.3)	
	Systemic JIA	26 (9.1)	17 (65.4)	9 (34.6)	
	Psoriatic	13 (4.5)	8 (61.5)	5 (38.5)	
	ERA	16 (5.6)	4 (25)	12 (75)	
	Undifferentiated	7 (2.4)	0	7 (100)	
	Do not know	82 (28.6)	10 (12.2)	72 (87.8)	
**Under systemic treatment**	Yes	205 (71.4)	71 (34.6)	134 (65.4)	**0.01**
	No	82 (28.6)	16 (19.5)	66 (80.5)	
**Fully vaccinated according to national vaccination schedule**	Yes	236 (82.2)	75 (31.8)	161 (68.2)	0.25
	No	46 (16)	12 (26.1)	34 (73.9)	
	Do not know	5 (1.7)	0	5 (100)	
**Past vaccine-related adverse event (any vaccine)**	Yes	38 (13.3)	3 (7.9)	35 (92.1)	**0.00**
	No	249 (86.7)	84 (33.7)	165 (66.3)	
**Previous influenza vaccine**	Yes	117 (40.9)	38 (32.5)	79 (67.5)	**0.00**
	No	170 (59.1)	8 (4.7)	162 (95.3)	
**Vaccine recommended by medical staff**	Yes	160 (55.7)	77 (48.1)	83 (51.9)	**0.00**
	No	127 (44.3)	10 (7.9)	117 (92.1)	

*Chi square test

Data regarding vaccination history were collected. Most patients (82.2%, n = 236) were fully vaccinated according to their national vaccination schedules, while 40.9% (n = 117) had received the influenza vaccine at least once previously (Table 1). Side effects from any previous vaccinations were reported by 13% (n = 38), predominantly fever or local reactions (Table 1).

**Fig. 1:**
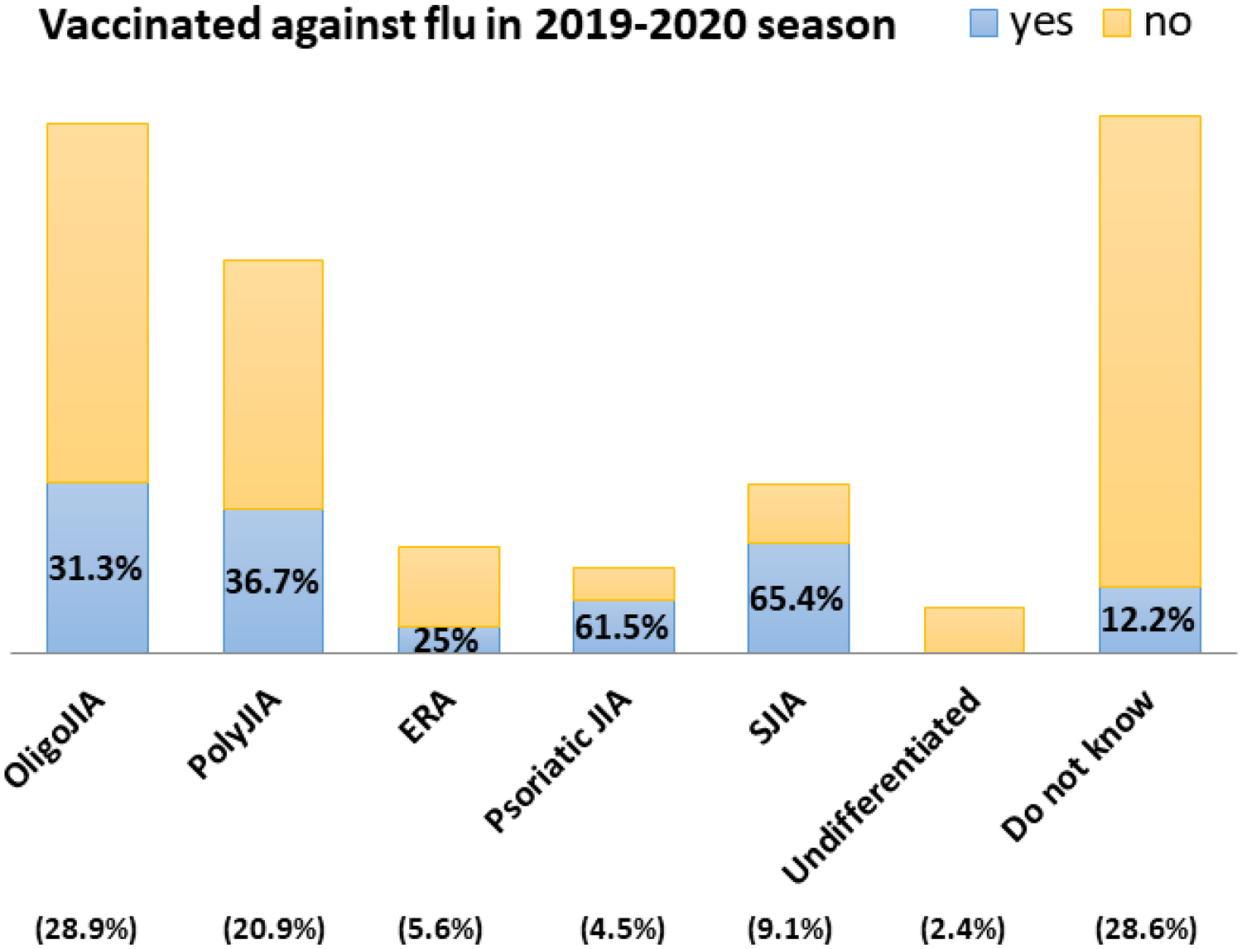
Influenza Vaccine Uptake for the different Juvenile Idiopathic Arthritis (JIA) types (X-axis includes the JIA type and its percentage out of the whole cohort. Y-axis represents the influenza vaccine uptake rate in each JIA subgroup)

With respect to influenza vaccine uptake in the 2019–2020 season, 87 children (30.3%) had been vaccinated against influenza. Disease status at the time of vaccination was stable in 78 (89.7%). The highest vaccine uptake was recorded in Greece (70.8%, n = 46), followed by Israel (41.9%, n = 26); none of the JIA patients from Croatia and Slovakia were vaccinated (p < 0.05; Table 1). Most of the 287 caregivers had been informed about the recommendation by a paediatric rheumatologist (33.4%, n = 96) or a paediatrician (27.9%, n = 80). Compared to employed caregivers, unemployed parents were more likely to vaccinate their children (25.7% vs. 53.3%, p < 0.05), while no significant differences in flu vaccine uptake were found with respect to marital status or education (Table 1). Children with systemic JIA had the highest vaccine uptake (65.4%, n = 17), while caregivers who did not know their child’s subtype of JIA reported the lowest uptake (12.2%, n = 10; p < 0.05) (Table 1, Fig. 1).

Furthermore, children receiving systemic treatment were more likely to have been vaccinated (34.6%, n = 71) in comparison with children who were not taking any medication (19.5%, n = 16; p < 0.05). Caregivers who were informed of influenza vaccine recommendations by medical staff and had vaccinated their children in the past were more likely to vaccinate in the 2019–2020 season (48.1%, n = 77 and 32.5%, n = 38, respectively), as compared to those who were not informed (7.9%, n = 10) or had not previously vaccinated their children (4.7%, n = 8; both p < 0.05). Notably, 38 patients who had previously experienced adverse vaccine-related events from any vaccine, reported the lowest vaccine uptake (7.9%, n = 3; p < 0.05).

**Fig. 2:**
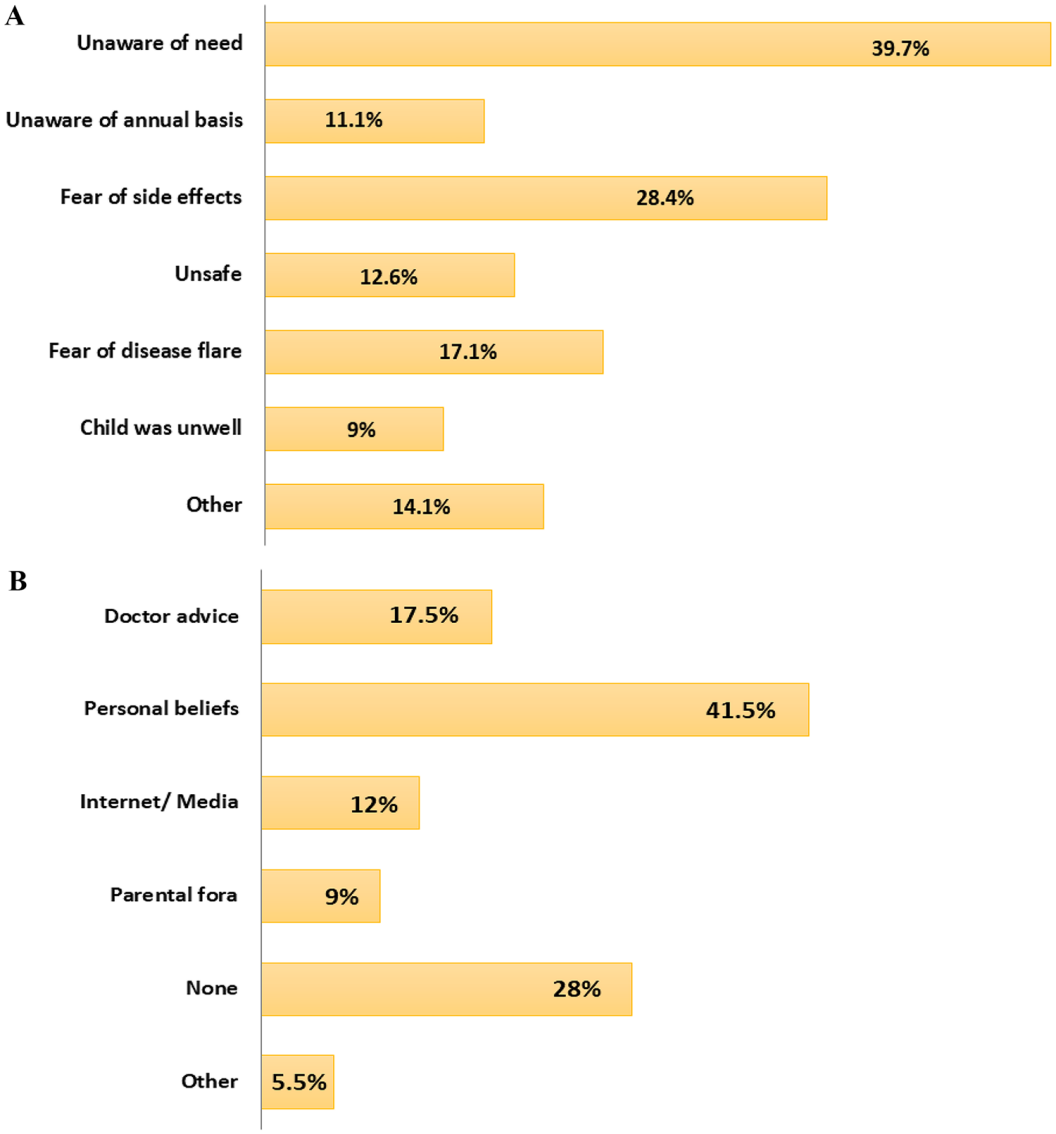
**A** Reasons for non-vaccination (some caregivers gave more than one answer). **B** Sources of information that led to non-vaccination (some caregivers gave more than one answer)

Among non-vaccinators, 60% (n = 119) reported not having had the opportunity to discuss their concerns with a specialist. The major reasons for non-vaccination included unawareness of the need (39.7%, n = 79), fear of side effects (28.4%, n = 57), and fear of disease flare (17.1%, n = 34) (Fig. 2A).

**Fig. 3:**
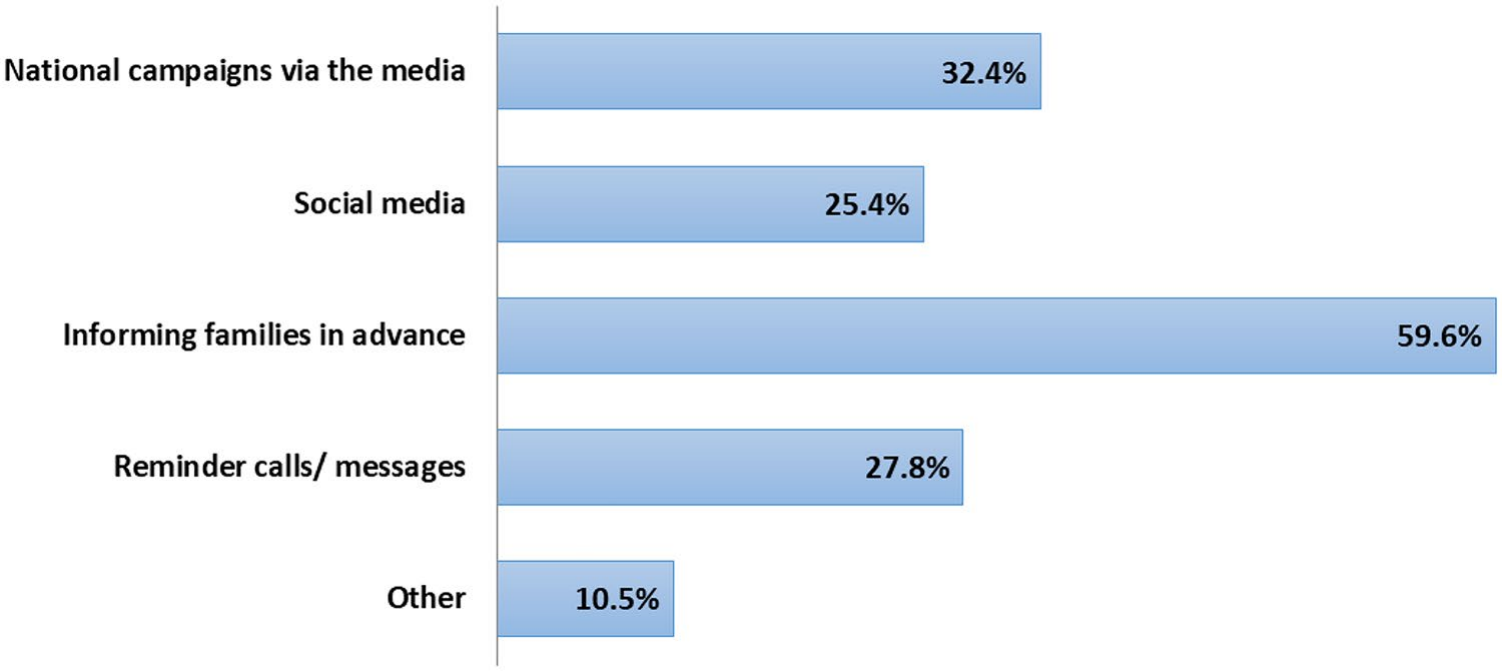
Suggestions of caregivers to improve influenza vaccine uptake in children with JIA (some caregivers provided more than one answer)

Personal beliefs primarily drove the decision for non-vaccination (41.5%, n = 83), while 17.5% (n = 35) reported that it was based on a doctor's advice (Fig. 2B). Among suggestions to improve influenza vaccine uptake in the future, “informing families in advance” was the most frequently cited recommendation from caregivers (59.6%, n = 171), followed by “organizing national campaigns” (32.4%, n = 93; Fig. 3).

## Discussion

Children with RD face increased susceptibility to infections, including influenza, compared to healthy individuals, mainly due to the underlying disease and immunosuppressive treatments [3]. Given the higher risk of severe influenza complications, annual vaccination is of paramount importance to protect this vulnerable population. Despite growing knowledge regarding the safety and immunogenicity of the influenza vaccine among patients with JIA [10], data regarding vaccine uptake is limited. To the best of our knowledge, this study represents the first multi-centre effort to assess influenza vaccination coverage in a JIA cohort and aims to identify barriers to vaccination and strategies to enhance vaccine uptake.

We found that only one-third of JIA patients received the influenza vaccine in the 2019–2020 season. Notable differences were found between different countries with a range of 0–70.8%. However, with the exception of Greece, vaccination rates in all countries were relatively low, falling within the 0% to 42% range.

Tuckerman et al. examined flu vaccine uptake in children with special-risk medical conditions, including RD and found that about half of the participants had received the vaccine at least once [11]. A national survey of primary care paediatricians in Greece regarding immunization practices in children with RD revealed that most (82%) were in favour of annual flu vaccine administration for this target group [12]. Other studies conducted to estimate immunization rates against influenza in high-risk paediatric patients reported a broad range of 5.1% to 42%, over time [13, 14]. In addition, a study targeting paediatric RD found suboptimal coverage of 10.2% against flu [7]. Interestingly, the current study reported great variations in flu vaccine uptake among participating countries, despite the published EULAR recommendations. The reasons for these differences between the countries need to be further investigated but they are likely affected by local medical practices, availability of vaccination campaigns, attitudes of the populations towards vaccination in general, and many other factors. An unexpected outcome in our study was that unemployed parents were more likely to vaccinate their children compared to employed caregivers (53.3% vs. 25.7%, p < 0.05). In contrast to our findings, unemployment [15] and low economic income [16] were found to be associated with lower COVID-19 vaccine acceptance in adults. Similarly, unemployment of the father was detrimental to the vaccination status of Belgian adolescents [17]. The low number of unemployed caregivers in our study may have been too small to enable us to draw firm conclusions about the relationship between socio-economic status and vaccination uptake.

This study aimed to identify factors that contribute to higher vaccine uptake. Our findings show that parental knowledge of the child’s principal diagnosis is an important factor in the decision to vaccinate the child against influenza. Caregivers who were unaware of their child’s specific diagnosis (subtype of JIA) reported the lowest vaccine uptake. In contrast, children diagnosed with systemic JIA and those receiving systemic medications were more likely to have received the influenza vaccine. This may be explained by the severity of symptoms in children with systemic JIA as compared to other types of JIA, which may have led caregivers to be more mindful of the risk of severe influenza illness. Moreover, caregivers of patients who had received a flu shot in the past felt more comfortable to have their children re-vaccinated in the 2019–2020 season. Previous experience with influenza vaccination and its safety and efficacy seem to be important considerations in the decision to obtain an annual vaccination.

Caregivers who were informed of the recommendation for seasonal influenza vaccine by medical staff, especially by a paediatrician or a paediatric rheumatologist, were more likely to vaccinate their children compared to those who were not informed. Our finding regarding the positive impact of medical professionals in promoting vaccinations is consistent with other studies [11, 13, 14, 18]. Caregivers may have more confidence in these specialists and perceive them to have more thorough knowledge and familiarity with their child’s condition. Therefore, it is reasonable to suggest providing vaccinations during outpatient paediatric rheumatology clinic visits, administered by the clinic nurse, as a strategy to enhance vaccination uptake.

On the other hand, caregivers who did not have the opportunity to discuss their concerns regarding the influenza vaccine with a specialist were hesitant to vaccinate their children. This underscores the importance of providing adequate time during appointments to discuss the safety and efficacy of annual flu vaccination with families.

Another barrier to vaccination was experiencing a vaccine-related adverse event. Caregivers whose children had previously experienced a systemic or even a local reaction after influenza vaccination were reluctant to revaccinate them. Other reasons we identified for vaccine hesitancy have been previously described, including concerns about vaccine safety and unawareness of the need for vaccination [14, 19].

There were some limitations to this study. Although the data regarding vaccine uptake was self-reported, caregivers’ reports of vaccine uptake should be reliable, especially when asked in the same year of vaccination. Another limitation is the relatively small number of JIA patients per country. However it is important to note that this research is a collaborative effort among pediatric rheumatologists from seven different countries and is not intended to encompass the entire population of children with JIA across these nations. Instead, our primary goal is to assess the overall influenza vaccine coverage among children with JIA. The multicenter nature of this collaboration allowed us to explore variations between countries. While we concur that the patient count from each country was comparatively modest, the collective sample of JIA parents/patients was substantial and enabled meaningful analysis. Another limitation was the variable proportion of JIA subtypes from the participating countries, as disease type and severity seemed to affect vaccination uptake. In addition, we noticed a large amount of educated caregivers in the cohort and we acknowledge the potential for selection bias in this study, as with any study relying on voluntary participation. However, the questionnaire was systematically offered and distributed to every JIA patient visiting the participating pediatric rheumatology clinics during the study period, which followed the influenza vaccination season. We observed a notably high acceptance rate among JIA patients' parents to participate and complete the questionnaire. Moreover, parents who preferred or needed help in reading the questionnaire and/or filling it out, received it from a research assistant that was reading the questions for them and filling the paperwork. Therefore, parents from all levels of education who came to clinic could participate.

It is worth noting that these data were collected before the COVID-19 pandemic. Our data show that many factors influenced vaccination rates across the different countries, even prior to the pandemic. These pre-pandemic data provide important information for future studies which will likely include the effect of COVID-19 on the process of decision-making for or against vaccination. Considering the impact of the SARS-CoV-2 crisis on attitudes and behaviours toward vaccination, it will be interesting to examine whether the views of parents/caregivers have changed since then.

## Conclusions

Despite variations among European countries, influenza vaccine uptake remains suboptimal among JIA patients, leaving a significant proportion of this vulnerable population unprotected against severe influenza illness. Our study showed that factors such as previous experiences with vaccination and information provided by medical professionals play essential roles in vaccine uptake. Informing families in advance to enable time to address their concerns, enhancing communication with medical specialists, and organizing targeted national campaigns to highlight the importance of the influenza vaccine can potentially lead to increased vaccination rates among children with JIA and other rheumatic diseases; thereby, contributing to improved health outcomes.

## Data Availability

No datasets were generated or analysed during the current study.

## Abbreviations:

EULAR: European league against rheumatism
Flu: Influenza
JIA: Juvenile idiopathic arthritis
PReS: Paediatric Rheumatology European Society
RD: Rheumatic diseases
